# Incidence of anogenital warts in Liuzhou, south China: a comparison of data from a prospective study and from the national surveillance system

**DOI:** 10.1038/emi.2017.100

**Published:** 2017-12-20

**Authors:** Feixue Wei, Wei Sheng, Xin Wu, Kai Yin, Jian Lan, Yue Huang, Xinjing Ma, Ya Zheng, Sijie Zhuang, Shoujie Huang, Yingying Su, Mingqiang Li, Ting Wu, Jun Zhang, Ningshao Xia

**Affiliations:** 1 State Key Laboratory of Molecular Vaccinology and Molecular Diagnostics, National Institute of Diagnostics and Vaccine Development in Infectious Diseases, Strait Collaborative Innovation Center of Biomedicine and Pharmaceutics, School of Public Health, Xiamen University, Xiamen 361102, Fujian, China; 2 Liuzhou Center for Disease Control and Prevention, Liuzhou 545027, Guangxi, China

**Keywords:** anogenital warts, human papillomavirus, incidence, prevalence, risk factors

## Abstract

To determine the incidence of anogenital warts (AGWs) in the Chinese general population, we compared the data from a prospective study and from the National Notifiable Disease Report System (NNDRS). A cohort study including 2378 women and 2309 men aged 18–55 years old enrolled from Liuzhou, China, was conducted with three scheduled visits at 6-month intervals from May 2014 to March 2016. And, a questionnaire survey was performed to collect the diagnosis history of AGWs at the enrollment visit. The data on reported AGW cases of Liuzhou in the NNDRS from 2006 to 2015 were also analyzed. Overall, the incidence rates of AGWs in the prospective study, in the self-reported diagnosis during past 12 months and in the NNDRS were 1.26 per 1000 person-years (95% confidence interval (CI): 0.16–2.37), 2.35 (95% CI: 1.17–4.20) and 0.183 (95% CI: 0.178–0.187), respectively. Human papillomavirus 6 or 11 were found in all the AGW biopsy samples (10/10). The onset time of AGWs in women was earlier, and the cumulative risk increased more quickly at a young age along with each subsequent younger birth cohort (*P*<0.0001), whereas slight differences were observed in the different male birth cohorts (*P*=0.0785). The sexual behavior of individuals and their sexual partners had a strong relationship with self-reported AGWs. Our study indicates that the incidence of AGWs in China is as high as that in developed countries, and the data based on the national surveillance system seriously underestimate the real disease burden of AGWs.

## INTRODUCTION

Anogenital warts (AGWs), also called genital warts or condyloma acuminata, is a common sexually transmitted disease (STD) in both men and women.^1^ Patients with AGWs suffer both physical (burning, bleeding, itching and pain) and psychosocial stress (anxiety, decreased self-esteem, and negative emotional and sexual impact).^2, 3, 4^ The recurrence of AGWs is common, with the rate ranging from 6% to 77%, which has further increased the economic burden of medical health care and the psychosocial impact on patients.^5, 6^ Previously, it was thought that AGWs were benign and not life threatening; however, recently, a Danish study indicated that individuals with AGWs had a long-term increased risk of anogenital cancers and head and neck cancers, which may due to coinfection with oncogenic human papillomavirus (HPV), having high-risk behavioral factors (e.g., a higher number of partners, or a higher consumption of alcohol or smoking) or the poor immunity of the patients.^7^

The incidence rates of AGWs among the general population worldwide are 1.6–2.8 per 1000 person-years,^8^ and the higher rates, over 10 per 1000 person-years, were reported in 15–26-year-old people,^9, 10^ sex workers,^11^ men who have sex with men^12, 13^ and HIV-infected individuals.^13^ In China, data regarding the incidence rate of AGWs mainly come from the National Notifiable Disease Report System (NNDRS); however, as AGWs is not a mandatory reported disease, many AGW cases are missed. The reported incidence rates from the NNDRS, which were 0.24–0.30 per 1000 person-years from 2008 to 2016, might severely underestimate the real disease burden of AGWs in China,^14^ and data from the general population based on cohort studies are scarce.

HPV types 6 and 11 cause more than 90% of the cases of AGWs.^15^ The administration of HPV vaccines containing HPV 6 and 11 antigens is the highly effective approach for preventing AGWs. After the introduction of the national quadrivalent HPV (types 6, 11, 16 and 18, Gardasil) vaccination program, a significant decline in diagnosed AGWs at the population level has been reported in Australia,^16, 17^ Denmark^18, 19, 20^ and the United States.^21^ Further, a recent ecological study in England, where a bivalent HPV vaccine (types 16 and 18, Cervarix) program was introduced in 2008, has shown a reduction in the rates of AGW diagnoses in males and females (25.4% and 30.6%, respectively) from 2009 to 2014.^22^ Gardasil and Cervarix were licensed in China in May 2017 and July 2016, respectively. Uncovering the real incidence rate of AGWs in China before HPV vaccination is essential to understand the effectiveness of the licensed HPV vaccines in China.

The purpose of this study was to investigate the prevalence and incidence of AGWs in the Chinese general population through the comparison of data from a prospective study and from the national surveillance system. In addition, we estimated the risk factors associated with self-reported history of clinically diagnosed AGWs.

## MATERIALS AND METHODS

### Data sources

#### The prospective study

From May to July 2014, 2309 males and 2378 females aged 18–55 years were enrolled from the general population via media advertising, flyers, posters and educational presentations in Liuzhou City, Guangxi, China. The details of the selection criteria of the population and the methods of testing have been described elsewhere.^23^ Volunteers were requested to attend three scheduled visits at 6-month intervals for 1 year to investigate the natural history of AGWs. At each visit, the volunteers underwent a clinical examination by trained clinicians to identify whether there were AGWs lesions in cervical, vaginal, vulvar, anal and perianal sites of women or in external genital, anal and perianal sites of men. For cases that were diagnosed with AGWs based on their clinical appearance, biopsy samples were obtained to be tested for HPV DNA and genotyped by PCR assay. The kits tested for 13 oncogenic HPV types (HPV 16, 18, 31, 33, 35, 39, 45, 51, 52, 56, 58, 59 and 68) and 3 non-oncogenic HPV types (HPV 6, 11 and 66). The study was approved by the Ethics Committee of Liuzhou Center for Disease Control and Prevention (CDC). Informed consent was given by all study volunteers.

At the enrollment visit, a questionnaire survey was conducted by a trained interviewer to collect information about ever having had a diagnosis of AGWs, the age at first diagnosis of AGWs, AGWs during the previous 12 months, ever having had a recurrence of AGWs and ever having sexual partners with previous episodes of STDs, including trichomoniasis, genital chlamydial infection, genital herpes, syphilis, gonorrhea and chancroid. Furthermore, we collected information regarding sociodemographic variables, hygienic habits and sexual behaviors with the questionnaire.

#### The national surveillance system

The NNDRS is a real-time, web-based, disease surveillance system that monitors 36 notifiable and non-notifiable diseases in China. It was established in 2003 after the outbreak of Severe Acute Respiratory Syndrome. It consists of a five-level network from the township to the national level and a three-level platform from prefecture to the national level. Infectious diseases are reported to the local disease reporting system within 2 h (class A infectious diseases) or 24 h (class B or C infectious diseases) post diagnosis in hospitals or other health facilities. To ensure the quality of the reports, the data are reviewed by the local CDC, then a correction or supplementary report is made if necessary.

AGWs is one of the non-notifiable diseases, and is monitored as one of the STDs according to the National Guidelines of Surveillance for Sexually Transmitted Diseases by the NNDRS in China. According to the guidelines, only the first episode patients were reported, whereas the return visit patients and recurrence patients were not reported. The cases were diagnosed according to the Diagnostic Criteria and Principles of Management of Condyloma Acuminatum (WS235–2003). The data of AGWs in Liuzhou City from 1 January 2006 to 31 December 2015 from the NNDRS were analyzed. Local population data by age, group and sex between 2006 and 2015 were obtained from the Liuzhou Statistics Department.

### Statistical analysis

#### The prospective study

The participants who were free of AGWs at baseline and who had at least one follow-up visit were included in the analysis for the prospective study. Pearson's *χ*^2^ tests were used to compare the sociodemographic, hygiene and sexual behavioral characteristics of men and women in the analyzed cohort and the full cohort. The incidence of AGWs was estimated by dividing the number of cases by the number of person-years of participants. Person-years were regarded as the number of years from the date of enrollment until the date that AGWs were observed or until the date of the last visit for those who did not develop AGWs. The calculation of the 95% confidence intervals (CIs) of the incidence was based on the number of events modeled as a Poisson variable for the total person-years. In addition, we calculated the prevalence of AGWs in the full cohort at baseline.

For data from the questionnaire survey, we assessed the incidence of AGWs during the previous 12 months by sex. The proportion of volunteers who had self-reported AGWs during a lifetime was also estimated. Based on the age at first diagnosis of AGWs, the Kaplan–Meier method was used to calculate the cumulative age-specific incidence, with strata by birth cohort (i.e., birth in 1959–1968, 1969–1978, 1979–1988 and 1989–1996). Comparisons among different birth cohorts were performed by the log-rank test. Odds ratios (ORs) and the corresponding 95% CIs of the risk factors associated with the self-reported AGWs were calculated by bivariate and multivariate logistic regression. Variables with *P*<0.20 in the bivariate analysis were put into the multivariate model and then selected stepwise, with a 0.05 significance level for entry and 0.05 for removal.

#### The national surveillance system

The incidence rates of AGWs by sex in NNDRS were calculated by using the total number of cases during the annual period divided by the local annual population at the time at risk. Temporal trends in the incidence from 2006 to 2015 were examined by fitting joinpoint models. The annual percentage change (APC) was used to express the trends, and the *Z*-test was used to assess whether the APC was significantly different from zero. To clarify the age distribution of the incidence by sex, we also calculated the average age-specific incidence rate of men and women.

To compare data from different sources, the incidence rates were reported per 1000 person-years in both the prospective study and the NNDRS. In this study, we used SAS version 9.4 (SAS Institute, Cary, NC, USA) to analyze the data, and *P*<0.05 was considered statistically significant.

## RESULTS

### The prospective study

A total of 3866 individuals, including 1821 men and 2045 women who were free of AGWs at baseline and who had at least one follow-up visit, were included in the analyzed cohort. Men and women in the analyzed cohort and the full cohort were comparable for most of the demographic and behavioral characteristics, excluding birth cohort and marital status (Table 1).

The median follow-up time was 12.5 months (interquartile range 12.1–13.1) for males and 12.6 months (interquartile range 12.5–13.0) for females. During the follow-up period, one female at Visit 2 and four females at Visit 3 developed AGWs (Table 2), and the incidence rate was 2.37/1000 person-years (95% CI: 0.30–4.45) (Table 3). None of the males was diagnosed with AGWs during follow-ups in the study period. At baseline, two (0.09%) males and five (0.21%) females were diagnosed with AGWs. Regardless of prevalent or incident cases, no recurrences were observed after treatment.

Because of loss to follow-up, we collected biopsy samples from 83% (10/12) of the prevalent and new incident AGW cases (Table 2). All samples were positive for HPV 6 or 11, and four samples were coinfected with HPV 16 or 18.

For all volunteers in the full cohort, 1.73% (40/2309) of the males and 1.14% (27/2378) of the females reported having at least one episode of clinically diagnosed AGWs during their lifetime (Table 3). Females generally acquired AGWs somewhat earlier than males (median age=26 years for females and 30 years for males), although no significant difference by sex was found (*P*=0.1108). Among the patients, 12.5% of the males and 18.5% of the females reported that they had at least one recurrence of AGWs. The incidence of self-reported, diagnosed AGWs during the past 12 months was 1.73 (95% CI: 0.36–3.42) per 1000 person-years for males and 2.94 (95% CI: 0.77–5.12) per 1000 for females, and no sex differences were found.

**Figure 1:**
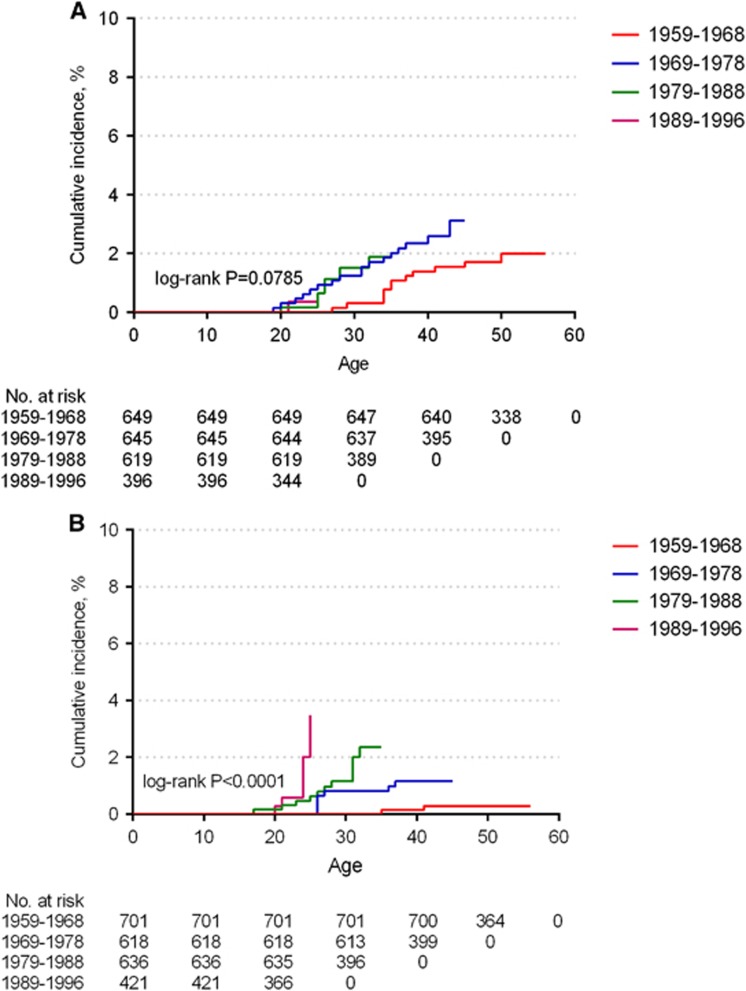
Kaplan–Meier estimates of the cumulative incidence of self-reported clinically diagnosed anogenital warts by birth cohort in the prospective study in men (**A**) and women (**B**). Based on age at first diagnosis of anogenital warts, the cumulative incidence of anogenital warts (up to that time) was calculated. The log-rank test was used to analyze the differences among different birth cohorts.

Figure 1 shows the cumulative incidence of self-reported diagnosed AGWs among men and women by birth cohort based on age at first diagnosis. In men, the cumulative incidence of AGWs was comparable among the different birth cohorts (*P*=0.0785); however, in women, the age at first diagnosis was earlier, and the cumulative incidence risk increased with each subsequent younger birth cohort (*P*<0.0001).

We estimated the risk factors associated with self-reported diagnosis of AGWs (Table 4). In the multivariate analysis, individuals whose sexual partners having a STD diagnosis (OR=4.3, 95% CI: 2.4–7.5) and who had more than three lifetime sexual partners (compared with 1, OR=4.1, 95% CI: 2.1–7.9) had higher risk to reported AGWs. In addition, sexual partners having sex with others (OR=2.4, 95% CI: 1.2–5.1) and having a sauna (OR=2.0, 95% CI: 1.2–3.4) increased the probability of self-reported AGWs.

### The national surveillance system study

**Figure 2:**
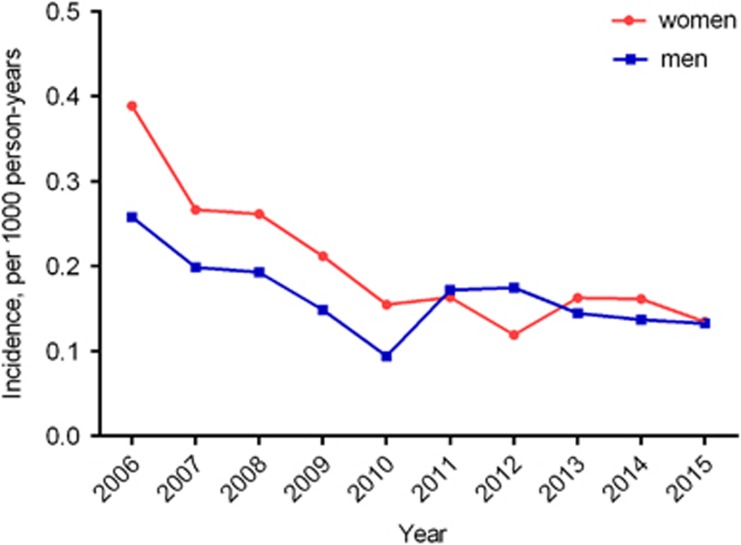
The temporal trend in incidence rate (/1000 person-years) of anogenital warts by sex from 2006 to 2015 in the national surveillance system.

A total of 3245 males and 4430 females in Liuzhou City were diagnosed with AGWs in the NNDRS from 2006 to 2015. The average incidence was 0.165 per 1000 person-years in men and 0.202 per 1000 in women (Table 3). The annual AGWs incidence decreased from 0.26 to 0.13 per 1000 person-years among males from 2006 to 2015 (APC=−6.20, *P*<0.01). Among females, it decreased from 0.39 to 0.15 per 1000 during 2006 to 2010 (APC=−19.64, *P*<0.01), then the stable trend was seen from 2010 to 2015 (APC=−2.41, *P*=0.70) (Figure 2).

**Figure 3:**
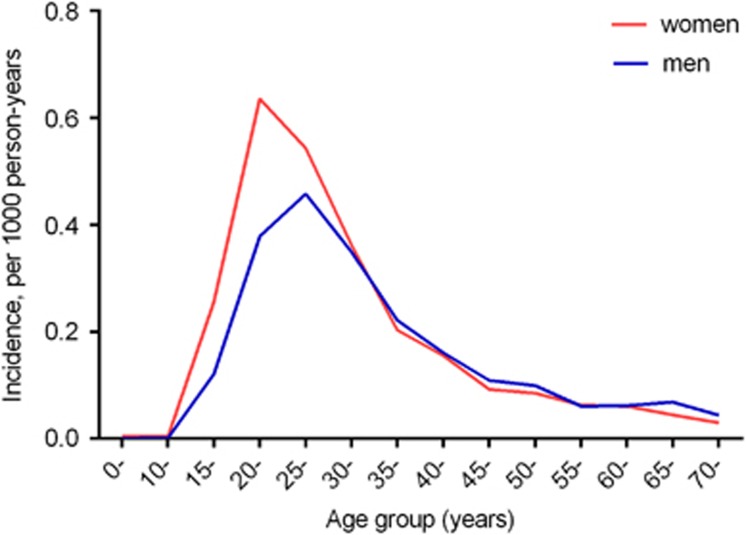
The average age-specific incidence rate (/1000 person-years) of anogenital warts by sex in the national surveillance system.

The average age-specified incidence rate of AGWs was highest for 20–24-year-old females (0.64/1000 person-years), while the rate peaked at a slightly older age of 25–29 years in males (0.46/1000 person-years) (Figure 3). Most of the AGW cases in the NNDRS were reported from the People’s Hospital and the Maternal and Child Health Care Hospital of Liuzhou, accounting for 58%, with no visible fluctuations over time.

In addition, we checked whether the self-reported AGWs occurred from 2006 to 2015 and the observed AGWs in our prospective study were reported to the NNDRS, and we found that the percentage was only 17% (6/35).

## DISCUSSION

The prevalence of AGWs in the general population in China was ~0.1–0.2%, and the incidence was 2 per 1000 person-years in the prospective study. HPV 6 or 11 were found in all of the AGWs biopsy samples. Females had an earlier peak incidence of AGWs than that did males, and the sexual behavior of women started earlier in younger birth cohorts. The lifetime number of sexual partners and the sexual behavior of sexual partners had strong relationships to self-reported AGWs.

Our data showed the incidence of AGWs in China was ~2/1000 person-years based on the prospective study, which is similar to the rate in Europe, North America, South America, Asia and Australia, where the annual incidence of AGWs ranged from 160 to 289 per 100 000;^8^ however, the reported incidence in the NNDRS was only about one-tenth of that of the prospective study, which is interestingly concordant with our finding that 17% of the AGW cases were reported to the NNDRS. The huge missing report rate can probably be explained by the following factors: first, in China, AGWs is a non-notifiable disease, and thus, reports of AGWs in the NNDRS were not carefully monitored and managed; and second, due to a variety of psychological and social reasons, patients with AGWs might not seek health care or may select private clinics that have no access to NNDRS for treatment. As decades are needed to observe the effectiveness of HPV vaccines against cervical cancers at the population level, the NNDRS for AGWs in China should be strengthened and monitored so that the effectiveness of HPV vaccines can be evaluated based on the incidence of AGWs over a short period.

In our study, the occurrence of HPV 6- or 11-positive AGWs lesions was 100% (10/10), and although the sample size was small, this finding implied that it would be effective to prevent AGWs by giving HPV vaccination against HPV 6 and 11 in China. Although younger and sexually active adults had the peak incidence, older adults remained at risk of acquiring AGWs and also needed to be vaccinated. In accordance with another study, coinfection with oncogenic types 16 and 18 was also found in subjects with AGWs.^24^ AGW cases coinfected with high-risk HPV types were at a higher risk of suffering anogenital cancers and head and neck cancers, so more attention should be paid to consecutively monitor these infections after treatment.^7^

Based on the self-reported AGWs during a lifetime, men had a higher prevalence than women; however, the reverse was observed in the prospective study and for the self-reported AGW cases during the past 12 months. In fact, it is a disputed issue whether there are sex-based discrepancies in the incidence of AGWs.^8^ The epidemiological studies, however, have consistently confirmed that women have an earlier peak incidence age than men.^25, 26, 27^ In our study, 20–25-year-old females and 25–30-year-old males had the highest incidence rate. This age-specific variation may be due to sexual mixing patterns, where younger women tended to have sex with older men. Another potential cause may be that females had a shorter incubation period of HPV 6 or 11 than men. After the incident HPV 6 or 11 infection, the median time of developing AGWs was 2.9 months in women, whereas it was 11 months in males.^28, 29^ The longer period in men may be the reason why no male cases were observed during the 1-year period of our prospective study.

Based on the analysis of different birth cohorts by sex, we found that the onset time of AGWs in women was earlier, and the cumulative risk increased more quickly at a young age along with each subsequent younger birth cohort, whereas only slight differences occurred in the different male birth cohorts. Future studies with larger sample size should take into consideration to interpret the sex differences; however, the phenomenon in the present study revealed that the main target population of the HPV vaccine should be carefully selected to make sure it is given before sexual debut, especially for women. For men, the vaccination of older birth cohorts might be warranted because of the stable cumulative risk of AGWs with age.

Similar to most studies, we found that sexual behavior was associated with self-reported clinically diagnosed AGWs.^30, 31, 32^ As the CDC recommends, altering sexual behavior by limiting the number of sexual partners and correctly using condoms are methods to avoid HPV infection.^33^ In addition, we found that ever having used a sauna was also associated with contracting AGWs, which reflected that developing good health habits could reduce the risk of incident AGWs.

Limitations existed in our study. First, as volunteers were enrolled in this study, the results presented here might have bias when generalized to the whole population in China. Second, although one-to-one questionnaire interviews in an independent room were conducted by a same-sex trained investigator, social desirability bias might occur when we collected the information about sexual behavior and other factors. Furthermore, the follow-up period is only 1 year in this study, which is similar to the median time from incident HPV 6 or 11 infection to the lesion development among males,^28, 29^ coupled with that men who contracted AGWs are more easily to recognize the lesions and refuse to continue the visits. Therefore, our perspective study may underestimate the incidence of AGWs in men.

In conclusion, the incidence of AGWs in China is as high as that in developed countries, and the data based on the national surveillance system seriously underestimate the real disease burden of AGWs in China.

## Figures and Tables

**Table 1:** Baseline characteristics of men and women in the analyzed cohort and the full cohort in the prospective study

**Variables**	**Analyzed cohort**^a^		**Full cohort**	
	**Men (*****n*****=1821)**	**Women (*****n*****=2045)**	**Men (*****n*****=2309)**	**Women (*****n*****=2378)**
*Birth cohort (year)*^b^				
1989–1996	256 (14.1)	295 (14.4)	396 (17.2)	421 (17.7)
1979–1988	458 (25.1)	537 (26.3)	619 (26.8)	638 (26.8)
1969–1978	532 (29.2)	567 (27.7)	645 (27.9)	618 (26.0)
1959–1968	575 (31.6)	646 (31.6)	649 (28.1)	701 (29.5)

*Region*				
Rural	747 (41.0)	1166 (57.0)	894 (38.7)	1391 (58.5)
Urban	1074 (59.0)	879 (43.0)	1415 (61.3)	987 (41.5)

*Education (years)*				
<12	1454 (79.8)	1684 (82.3)	1837 (79.6)	1956 (82.3)
≥12	367 (20.2)	361 (17.7)	472 (20.4)	422 (17.7)

*Income (CNY)*				
<50 000	1364 (74.9)	1620 (79.2)	1733 (75.1)	1890 (79.5)
≥50 000	457 (25.1)	425 (20.8)	576 (24.9)	488 (20.5)

*Marital status*^b^				
Married/cohabitating	1435 (78.8)	1756 (85.9)	1732 (75.0)	1967 (82.7)
Single/divorced/separated/widowed	386 (21.2)	289 (14.1)	577 (25.0)	411 (17.3)

*Ever having a sauna*				
No	1219 (66.9)	1510 (73.8)	1566 (67.8)	1747 (73.5)
Yes	602 (33.1)	535 (26.2)	743 (32.2)	631 (26.5)

*Ever used a towel supplied by the sauna*				
No	1309 (71.9)	1653 (80.8)	1676 (72.6)	1906 (80.2)
Yes	512 (28.1)	392 (19.2)	633 (27.4)	472 (19.8)

*Ever stayed in a hotel*				
No	323 (17.7)	707 (34.6)	391 (16.9)	807 (33.9)
Yes	1498 (82.3)	1338 (65.4)	1918 (83.1)	1571 (66.1)

*Ever used a towel supplied by the hotel*				
No	702 (38.6)	1299 (63.5)	871(37.7)	1472(61.9)
Yes	1119 (61.4)	746 (36.5)	1438(62.3)	906(38.1)

*Age at time of first sexual intercourse*				
<18 years	130 (7.1)	108 (5.3)	190 (8.2)	145 (6.1)
18–25 years	1391 (76.4)	1720 (84.1)	1769 (76.6)	1999 (84.1)
≥26 years	300 (16.5)	217 (10.6)	350 (15.2)	234 (9.8)

*Lifetime number of sexual partners*				
1	847 (46.5)	1372 (67.1)	1040 (45.0)	1554 (65.4)
2–3	563 (30.9)	592 (28.9)	731 (31.7)	726 (30.5)
≥4	411 (22.6)	81 (4.0)	538 (23.3)	98 (4.1)

*Number of sexual partners in past year*				
0–1	1553 (85.3)	1963 (96.0)	1962 (85.0)	2270 (95.5)
≥2	268(14.7)	82 (4.0)	347 (15.0)	108 (4.5)

*Sexual partners having sex with others*				
No	681 (37.4)	813 (39.8)	874 (37.9)	971 (40.8)
Yes	76 (4.2)	92 (4.5)	104 (4.5)	108 (4.5)
Unknown	1064 (58.4)	1140 (55.8)	1331 (57.6)	1299 (54.6)

*Previous STD diagnosis*				
No	1683 (92.4)	1693 (82.8)	2137 (92.6)	1984 (83.4)
Yes	138 (7.6)	352 (17.2)	172 (7.4)	394 (16.6)

*Frequency of condom use*				
Never	858 (47.1)	1142 (55.8)	1079 (46.7)	1275 (53.6)
Sometimes	566 (31.1)	579 (28.3)	712 (30.8)	698 (29.4)
Always	397 (21.8)	324 (15.8)	518 (22.4)	405 (17.0)

Abbreviations: Chinese Yuan, CNY; sexually transmitted disease, STD.

a Men and women who were free of anogenital warts at baseline and who had at least one follow-up visit.

b Significant difference between the analyzed cohort and full cohort at *P*<0.05 using *χ*^2^ test in both men and women.

**Table 2:** Characteristics of prevalent and incident patients in the prospective study

**ID**	**Sex**	**Age (years)**	**Onset time**	**Lesion sites**	**HPV types**
1	Female	50	Visit 1	Cervix	6
2	Female	34	Visit 1	Vulva	6, 11, 16 and 18
3	Female	32	Visit 1	Vulva	6
4	Female	27	Visit 1	Vulva	6 and 16
5	Female	28	Visit 1	Vulva and anal	6
6	Male	22	Visit 1	Anal	11
7^a^	Male	31	Visit 1	Penis	—
8	Female	52	Visit 2	Vulva	6
9	Female	38	Visit 3	Vulva	6, 11, 16 and 18
10	Female	22	Visit 3	Vulva	6, 11, 16 and 18
11	Female	21	Visit 3	Vagina	6
12^a^	Female	20	Visit 3	Vulva and perianal	—

Abbreviation: human papillomavirus, HPV.

a Biopsy samples of anogenital warts were not collected because of loss to follow-up.

**Table 3:** Prevalence and incidence of anogenital warts by sex based on data from the prospective study and from the national surveillance system

**Variables**	**Males**	**Females**	**Total**
*Prevalence (%, 95% CI)*			
Self-reported a lifetime history of AGWs in the prospective study	1.73 (1.24, 2.35)	1.14 (0.75, 1.65)	1.43 (1.11, 1.81)
Cases diagnosed at baseline in the prospective study	0.09 (0.01, 0.31)	0.21 (0.07, 0.49)	0.15 (0.06, 0.31)

*Incidence (/1000 person-years, 95% CI)*			
Follow-up data in the prospective study	0	2.37 (0.30, 4.45)	1.26 (0.16, 2.37)
Self-reported data during the past 12 months in the prospective study	1.73 (0.36, 3.42)	2.94 (0.77, 5.12)	2.35 (1.17, 4.20)
Data in the national surveillance system^a^	0.165 (0.159, 0.171)	0.202 (0.196, 0.209)	0.183 (0.178, 0.187)

Abbreviations: anogenital warts, AGWs; confidence interval, CI.

a Data show the average incidence of AGWs from 2006 to 2015.

**Table 4:** Factors associated with self-reported clinically diagnosed anogenital warts in the prospective study by bivariate and multivariate analysis

	***n*** **(%)**	**OR (95% CI)**	**AOR (95% CI)**^a^
*Birth cohort (year)*			
1989–1996	6 (0.7)	1.0	—
1979–1988	23 (1.8)	2.5 (1.0, 6.2)	—
1969–1978	24 (1.9)	2.6 (1.1, 6.4)	—
1959–1968	14 (1.0)	1.4 (0.5, 3.7)	—

*Sex*			
Female	27 (1.1)	1.0	—
Male	40 (1.7)	1.5 (0.9, 2.5)	—

*Ever having a sauna*			
No	28 (0.8)	1.0	1.0
Yes	39 (2.8)	3.4 (2.1, 5.6)	2.0 (1.2, 3.4)

*Lifetime number of sexual partners*			
1	17 (0.7)	1.0	1.0
2–3	21 (1.4)	2.2 (1.2, 4.2)	1.6 (0.8, 3.2)
≥4	29 (4.6)	7.2 (4.0, 13.3)	4.1 (2.1, 7.9)

*Sexual partners having sex with others*			
No	24 (1.3)	1.0	1.0
Yes	13 (6.1)	5.0 (2.5, 9.9)	2.4 (1.2, 5.1)
Unknown	30 (1.1)	0.9 (0.5, 1.5)	0.8 (0.4, 1.3)

*Sexual partners having a previous STD diagnosis*			
No	47 (1.1)	1.0	1.0
Yes	20 (7.5)	7.6 (4.4, 13.0)	4.3 (2.4, 7.5)

Abbreviations: sexually transmitted diseases, STD; odds ratio, OR; adjusted odds ratio, AOR; confidence interval, CI.

a Factors in the multivariate model were birth cohort, sex, income, ever having a sauna, ever used a towel supplied by the sauna, ever stayed in a hotel, ever used a towel supplied by the hotel, lifetime number of sexual partners, number of sexual partners in the past year, frequency of condom use, sexual partners having sex with others and sexual partners having a previous STD diagnosis.
